# Cilostazol attenuates intimal hyperplasia in a mouse model of chronic kidney disease

**DOI:** 10.1371/journal.pone.0187872

**Published:** 2017-12-05

**Authors:** Wiwat Chancharoenthana, Asada Leelahavanichkul, Sujittra Taratummarat, Jutamas Wongphom, Khajohn Tiranathanagul, Somchai Eiam-Ong

**Affiliations:** 1 Division of Nephrology, Department of Medicine, Faculty of Medicine, Chulalongkorn University, and King Chulalongkorn Memorial Hospital, Thai Red Cross Society, Bangkok, Thailand; 2 Section of Interventional Nephrology, Division of Nephrology and Hypertension, Department of Medicine, Chulabhorn Hospital, HRH Princess Chulabhorn College of Medical Science, Chulabhorn Royal Academy (CRA), Bangkok, Thailand; 3 Department of Microbiology, Faculty of Medicine, Chulalongkorn University, Bangkok, Thailand; 4 Center of Excellence in Immunology and Immune-mediated Diseases, Department of Microbiology, Chulalongkorn University, Bangkok, Thailand; 5 Department of Pathology, Faculty of Medicine, Chulalongkorn University, Bangkok, Thailand; Temple University School of Medicine, UNITED STATES

## Abstract

Intimal hyperplasia (IH) is a common cause of vasculopathy due to direct endothelial damage (such as post-coronary revascularization) or indirect injury (such as chronic kidney disease, or CKD). Although the attenuation of coronary revascularization-induced IH (direct-vascular-injury-induced IH) by cilostazol, a phosphodiesterase III inhibitor, has been demonstrated, our understanding of the effect on CKD-induced IH (indirect-vascular-injury-induced IH) is limited. Herein, we tested if cilostazol attenuated CKD-induced IH in a mouse model of ischemic-reperfusion injury with unilateral nephrectomy (Chr I/R), a normotensive non-proteinuria CKD model. Cilostazol (50 mg/kg/day) or placebo was orally administered once daily from 1-week post-nephrectomy. At 20 weeks, cilostazol significantly attenuated aortic IH as demonstrated by a 34% reduction in the total intima area with 50% and 47% decreases in the ratios of tunica intima area/tunica media area and tunica intima area/(tunica intima + tunica media area), respectively. The diameters of aorta and renal function were unchanged by cilostazol. Interestingly, cilostazol decreased miR-221, but enhanced miR-143 and miR-145 in either *in vitro* or aortic tissue, as well as attenuated several pro-inflammatory mediators, including asymmetrical dimethylarginine, high-sensitivity C-reactive protein, vascular endothelial growth factor in aorta and serum pro-inflammatory cytokines (IL-6 and TNF-α). We demonstrated a proof of concept of the effectiveness of cilostazol in attenuating IH in a Chr I/R mouse model, a CKD model with predominantly indirect-vascular-injury-induced IH. These considerations warrant further investigation to develop a new primary prevention strategy for CKD-related IH.

## Introduction

Intimal hyperplasia (IH) is a vasculopathy characterized by a differentiation of any cells that form a multilayer compartment at the tunica intima of blood vessels. IH is responsible for vascular complications in several chronic disorders including coronary artery disease (CAD), peripheral arterial disease (PAD), and chronic kidney disease (CKD) [1–3]. Direct endothelial damage is the most important factor that induced IH in CAD and PAD [3]. On the other hand, both direct vascular damage (hypertension) and indirect vascular injury (uremia, anemia and chronic inflammatory state) are responsible for IH in CKD [1, 4]. Because most animal studies on IH have been performed in experiments on a 5/6 nephrectomy model, or a model with chronic glomerulopathy with hypertension and heavy proteinuria, IH in these mice was influenced not only by CKD but also by the hypertension-associated direct endothelial injury. To see if the indirect-vascular-injury-induced IH of CKD exists without hypertension, another CKD model is needed. Interestingly, a CKD model with predominant tubulointerstitial damage, less albuminuria and hypertension is developed by ischemia-reperfusion injury with unilateral nephrectomy (Chr I/R) [5]. Hence, we examine indirect-vascular-injury-induced IH in this model.

In addition, previous studies demonstrate that several molecules are responsible for IH, including vascular endothelial growth factor (VEGF), platelet-derived growth factor (PDGF) [6–8], asymmetrical dimethylarginine (ADMA) [9], and several microRNAs (miRs) [10–12]. MiRs are small, noncoding RNAs of 18–22 nucleotides in length, which regulate posttranscriptional gene expression. There is growing evidence that indicates that miR is actively involved in the inflammatory processes and vascular IH progression [13, 14]. The linkage between miRs and IH severity implies the emerging role of miRs for IH monitoring. In general, miR-143, miR-145, and miR-221 are synthesized and secreted from endothelial cells (ECs) through extracellular vesicles to regulate vascular smooth muscle cell (VSMCs) functions [10]. Mir-221 overexpression enhances IH in a rat carotid artery balloon injury model [11], while miR-143 and miR-145 reduce IH severity in a rat carotid artery balloon injury model [12].

Thus, a therapeutic strategy with multiple effects might be a more appropriate treatment of IH. Indeed, cilostazol, a phosphodiesterase (PDE) III inhibitor, attenuates the direct vascular injury-induced IH through multiple mechanisms, including vasodilation and antiplatelet action [15], anti-inflammation and the reduction of platelet-leukocyte interaction [16] and the inhibition of vascular proliferation [17, 18] through the up-regulation of hepatocyte growth factors and enhancement of *p53* oncogene [19–21]. Moreover, cilostazol might modulate vascular-related growth factors and oxidative stress molecules such as VEGF [7], PDGF [8], and nitric oxides (NO) [22]. Nevertheless, the data on cilostazol’s effects on indirect-vascular-injury-induced IH is still limited. Therefore, we have conducted experiments to determine the therapeutic effects of cilostazol on indirect-vascular-injury-induced IH in a Chr I/R mouse model.

## Materials and methods

### Animal model

Thirty 6-week-old male CD-1 mice weighing 34±3 g were obtained from the National Laboratory Animal Center, Nakhon Pathom, Thailand. Animal care followed the National Institutes of Health criteria for the use and treatment of laboratory animals. The protocol for the experiment was approved by the Animal Care and Use Committee of the Faculty of Medicine, Chulalongkorn University (No.008/2556). The mice were separated to one animal per cage and maintained under conditions of 20–24°C at 40–70% humidity with a 12-hour light-dark cycle. Food and water were given *ad libitum*.

### Ischemic-reperfusion-injury-induced chronic kidney disease (Chr I/R) model

**Fig 1:**
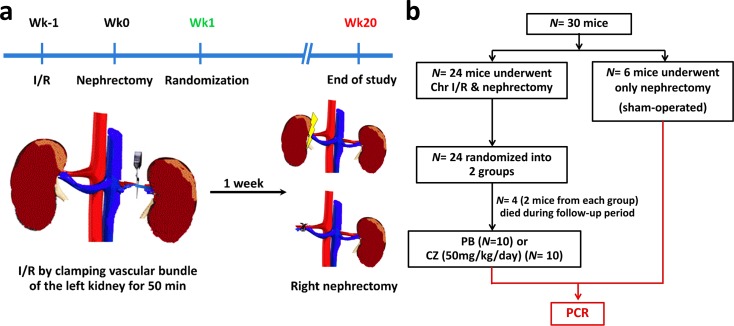
Overview of the study of the ischemic-reperfusion injury with unilateral nephrectomy (Chr I/R) mouse model. **(a)** Time-line of Chr I/R procedure and **(b)** the study schema was showed.

The procedure was performed in a 2-stage-operation under isoflurane anesthesia as previously described with some modifications [5]. In short, in the first week (wk-1), the left renal bundle was clamped through left flank incisions by atraumatic vascular clamp, and the incisions were closed layer by layer with nylon 2–0. The mice were then allowed to awaken in the box. Subsequently, the procedure was repeated to remove the clamps after a total ischemic time of 50 min. One week later (wk0), right nephrectomy was performed through right flank incisions (Fig 1A). For the sham-operated group, mice underwent left renal sham operations at wk-1, followed by right nephrectomy. The body temperature during all operations, including anesthesia, incision/clamping, and suturing was maintained by setting the temperature of the heating plate (Kleintier-OP-Tisch, Medax GmbH, Germany) to 37°C to avoid temperature effects on ischemic-reperfusion (I/R) injury [23]. At one week post-nephrectomy (wk1), Chr I/R mice were randomized to be administered either cilostazol (Pletaal^®^, PM129023, Otsuka, Tokushima, Japan) at 50 mg/kg/dose or a placebo control (reciprocal volume of normal saline) through daily oral gavage at 08:00 AM until euthanization at 20 weeks post-nephrectomy (wk20).

During the follow-up period, mice were monitored for clinical signs every day, including body condition, skin condition, estimated hydration, color of mucous membranes, heart rate and rhythm, respiratory rate, signs of diarrhea, and amount and appearance of urine and feces in the cage. Four mice died in wk15 and wk16 after randomization without preceding signs and symptoms. Therefore, 10 mice per group were available for analysis (Fig 1B). Body weights were monitored twice per week. Blood was drawn via tail vein at 1, 4, 12, 16 and 20 weeks post-nephrectomy. Serum samples were immediately centrifuged at 8,000*g* for 10 min at 4°C and kept at -80°C until used. Spot urine samples were collected every week for 4 consecutive weeks, then every 4 weeks, and kept at -80°C.

All mice were euthanized through cardiac puncture under isoflurane anesthesia, and internal organs (heart, lungs, and kidneys) were preserved in 10% neutral-buffered formalin for histological study. Despite the protective effect of isoflurane [24] and multiple isoflurane exposures, renal fibrosis was demonstrated in the Chr I/R mice model in both placebo- and cilostazol-treated mice. Aorta from the arch of aorta to iliac bifurcation was removed. The proximal one-third of the aortic arch was preserved in 10% neutral buffered formalin, while the distal two-thirds were preserved in RNAlater (ThermoFisher Scientific, Waltham, MA, USA) and stored at -80°C for miR study.

### Systolic blood pressure measurement

A tail cuff plethysmography (IITC Life Scientific Instruments, Woodland Hills, CA, USA) was used for the measurement of systolic blood pressure (SBP) as previously described elsewhere [25]. SBP was measured before the clamping of left renal bundle at wk-1 and then again at 4, 12, and 20 weeks after right nephrectomy. The measurements of SBP in the perioperative period were omitted due to the confounding variable of perioperative stress. SBP measurements were carried out several times before the start of the experiment to precondition the mice to the procedure. SBP measurements were obtained after the mice were familiar with the process. The means of three SBP measurements at each time point, with a 10-minute rest interval between the readings, were used to represent the data.

### Histology and assessment of glomerular and interstitial injuries

Kidney sections (3 μm thickness) were stained with hematoxylin and eosin (H&E), Periodic Acid-Schiff reagent (PAS), and Masson’s trichrome color. Glomerular injury was scored blindly by 3 pathologists. For glomerular injury scoring, 50 randomly selected glomeruli in each slide of PAS-stained sections were explored, and the sclerosis area in each glomerulus was assessed by a semi-quantitative scoring method: sclerotic area 0% = normal glomeruli; sclerotic area up to 25% = minimal sclerosis; sclerotic area 25 to 50% = moderate sclerosis; sclerotic area 50 to 75% = moderately severe sclerosis; and sclerotic area 75 to 100% = severe sclerosis. Quantitative analysis of interstitial compartments occupied by fibrotic tissue was defined by the relative interstitial volume of the total interstitium in each slide of Masson’s trichrome-stained sections. A standard point-counting method was used to quantitate the volume of the renal cortex [26]. The cortical region was analyzed in a stepwise fashion as a series of consecutive fields. For each field, a region of interest was traced that included cortical tubules and interstitial space. One point-count study evaluated interstitium only and was based on 1,200 points for each kidney. Glomeruli, large- or medium-sized blood vessels, and medullary tissue were excluded. The relative interstitial volume was scored as a percentage of affected renal parenchyma to total surface area of sampled cortical area. The average values of three consecutive measurements were used for all parameters.

### Histology and assessment of intimal hyperplasia

The vascular cross-sections of aorta (3 μm thickness) were stained with H&E and PAS, then quantitatively analyzed in ten sections per mouse, starting from a 5-μm^2^ area of the left ventricle. To minimize bias in measurement, the average values of three consecutive measurements were used for all parameters. Aorta thickness was measured by two pathologists with blinded experimental data by using Image-Pro® Premier 9.1 Software (Media Cybernetics Manufacturing, PA, USA). Lastly, the mean values of the scores of the two pathologists was taken. Tunica intima was an area inside internal elastic lamina and tunica media thickness was an area demarcated between internal and external elastic lamina. The tunica intima/tunica media ratio (I:M) and the ratio of tunica intima/(tunica intima + tunica media) (I:I+M) were used for the determination of IH.

### Blood and urine analysis

Blood urea nitrogen (BUN), serum creatinine (SCr), and urine creatinine (UCr) were determined by colorimetric assay (QuantiChrom Ureas and Creatinine kit, Bioassay, Hayward, CA, USA). Spot urine protein was measured by Bradford analysis (Bio-rad, Hercules, CA, USA), and proteinuria was presented with spot urine protein to urine creatinine ratio (UPCR; Spot urine protein/urine creatinine), an equivalent representative of 24h proteinuria in animal study [5]. Hematocrit (Hct) was assessed by micro-hematocrit method and Coulter Counter (Hitachi 917, IN, USA). ELISA assays were used for measuring ADMA (Enzo Life Sciences Inc., NY, USA), PDGF (Boster Biological Technology Co., Ltd., CA, USA), VEGF (Abcam, MA, USA), and high sensitivity C-reactive protein (hs-CRP) (Biocompare, CA, USA). Inflammatory cytokines, including interleukin (IL-6) and tumor necrosis factor (TNF-α), were measured by the Luminex Map-based multiplex technology using the MILLIPLEX MAP Mouse Cytokine Magnetic Bead Panel (Millipore, Billerica, MA, USA) on the Luminex instrument according to the manufacturer’s procedure. We measured ADMA, PDGF, VEGF, hs-CRP, IL-6, TNF-α and miRs (miR-143, miR-145, miR-221) at 4, 12, 16, and 20 weeks after nephrectomy.

## MicroRNA expression, *in vitro*

Primary Human Umbilical Vein Endothelial Cells (HUVECs: ATCC^®^ PCS-100-010^TM^) were used to test the effects of cilostazol on miR expression. HUVECs (100,000 cells/well) were plated into 24-well plates and incubated for 24 hours at 37°C in 5% CO_2_ atmosphere conditions. The cells were then treated with 3, 10, or 30 uM of cilostazol that were dissolved in dimethyl sulfoxide (DMSO) for 24 hours. For the control group, HUVECs were cultured in endothelial cell basal medium (EBM-2) (Lonza, Walkersville, MD, USA) complete medium containing 5% DMSO. After that, miRs were extracted by miRNeasy (Qiagen, Hilden, Germany). Quantification of miR-143, miR-145, miR-221, and RNU44 were measured using Taqman probes and real-time PCR (Applied Biosystems™ 7500). RNU44 was used as the house-keeping gene. The lists of primers (Invitrogen Life Technologies) were demonstrated as follows: primer RNU44 (ID001094):

5'CCTGGATGATGATAGCAAATGCTGACTGAACATGAAGGTCTTAATTAGCTCTAACTGACT 3', primer miR-145a (ID002278): 5' GUCCAGUUUUCCCAGGAAUCCCU 3', primer miR-143 (ID002249): 5' UGAGAUGAAGCACUGUAGCUC 3', primer miR-221 (ID002096): 5' ACCUGGCAUACAAUGUAGAUUU 3'. The expressions were determined by CT, and expression fold change was calculated as previously described elsewhere [27].

### RNA isolation and quantification

The protocol for this study followed a previous publication [28]. In brief, samples were placed into TRIzol (Invitrogen, Carlsbad, CA, USA), completely homogenized, and centrifuged at 10,000*g* for 15 min at 4°C. Next, the supernatant was put in glycogen 1 μL in chloroform 250 μL. Then, the samples were centrifuged again at 10,000*g* for 20 minutes at 4°C and supernatant was transferred into 1 mL of 70% ethanol and placed on an RNeasy column. Total RNA was evaluated by a miRNA easy kit (Qiagen, Valencia, CA, USA) for extraction. The samples were mixed again with denaturing buffer following the manufacturers’ protocols. Both qualification and quantification of miRs was performed with the NanoDrop ND-1000 spectrometer (NanoDrop Technologies, Wilmington, DE, USA).

### Reverse transcription, real-time PCR and graphical heat-map construction

Taqman probes for each gene and Taqman master mix were used to quantify synthetic miR spike-ins and cellular miR in real-time PCR assays according to the manufacturer’s protocols. Individual real-time PCR assays were performed in a 20 μL reaction volume on an ABI 7,500 real-time PCR system (Applied Biosystems, Waltham, MA, USA) based on the standard 7,500 run mode. The ΔΔCt method was used to calculate the relative expression (fold change) between sample groups. RNU6B expression was used for serum miR normalization [28]. For a better demonstration of miRs from aortic tissue in individual mice, a graphical heat-map construction of all RT-qPCR ΔCt values using Ward’s method was performed.

### Statistical analysis

All experimental data are expressed as mean±standard error (SEM). Analysis of variance (ANOVA) followed by *post hoc* Bonferroni’s correction was used for the analysis of more than 2 groups. A two-way repeated measure ANOVA was used when two or more time points were assessed. Correlations between variables were analyzed using Pearson’s correlation analysis, *p-*values of less than 0.05 were considered statistically significant. Statistical calculations were performed using SPSS for Windows 15.0 (SPSS for Windows; Chicago, IL) and GraphPad Prism 6.0 (GraphPad Software, La Jolla, CA, USA).

## Results

### Clinical manifestations of chronic kidney disease in ischemic-reperfusion injury with unilateral-nephrectomy-induced chronic kidney disease (Chr I/R) mice

**Fig 2:**
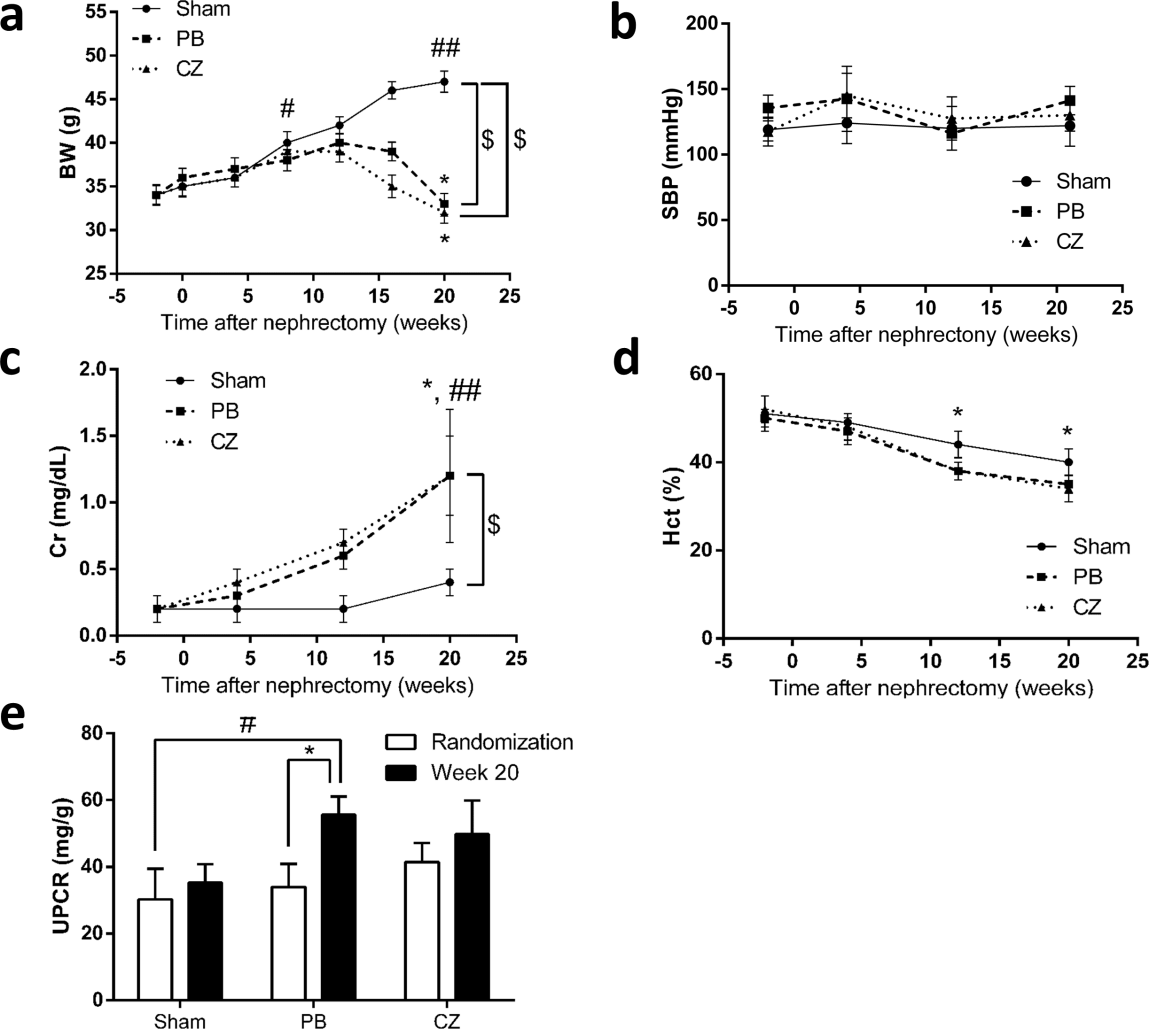
Clinical and laboratory manifestations of the ischemic-reperfusion injury with unilateral nephrectomy (Chr I/R) mouse model. **(a)** Body weight (BW), **(b)** systolic blood pressure (SBP), **(c)** serum creatinine (Cr), and **(d)** hematocrit (Hct) alteration of sham-operated (n = 6), placebo (PB) (n = 10), and cilostazol-treated mice (CZ) (n = 10/group) during the period of study were demonstrated in longitudinal assessment. **(e)** Quantitative proteinuria determined with urine protein creatinine ratio (UPCR) of Chr I/R mice at 1 week (randomization time) and at 20 weeks post-nephrectomy was showed. *p< 0.05 *vs* baseline in the same group; # *p*< 0.05, ## *p*< 0.01 *vs* baseline in sham-operated mice at wk-1; $ *p*< 0.05.

Clinical manifestations of CKD, including body weight (BW), systolic blood pressure (SBP), proteinuria, Hct, and urine volume, were explored in Chr I/R mice. CKD’s failure to thrive was demonstrated with the unchanged BW during the first 8 weeks post-nephrectomy in Chr I/R mice. In contrast, a significant weight gain was demonstrated in sham mice. At 20 weeks, there was a 6% decrease from the baseline BW in Chr I/R mice but a 38% increase in BW from the baseline in the sham mice (Fig 2A). Furthermore, the present Chr I/R mice model demonstrated no significant changes in SBP measurements (Fig 2B).

**Fig 3:**
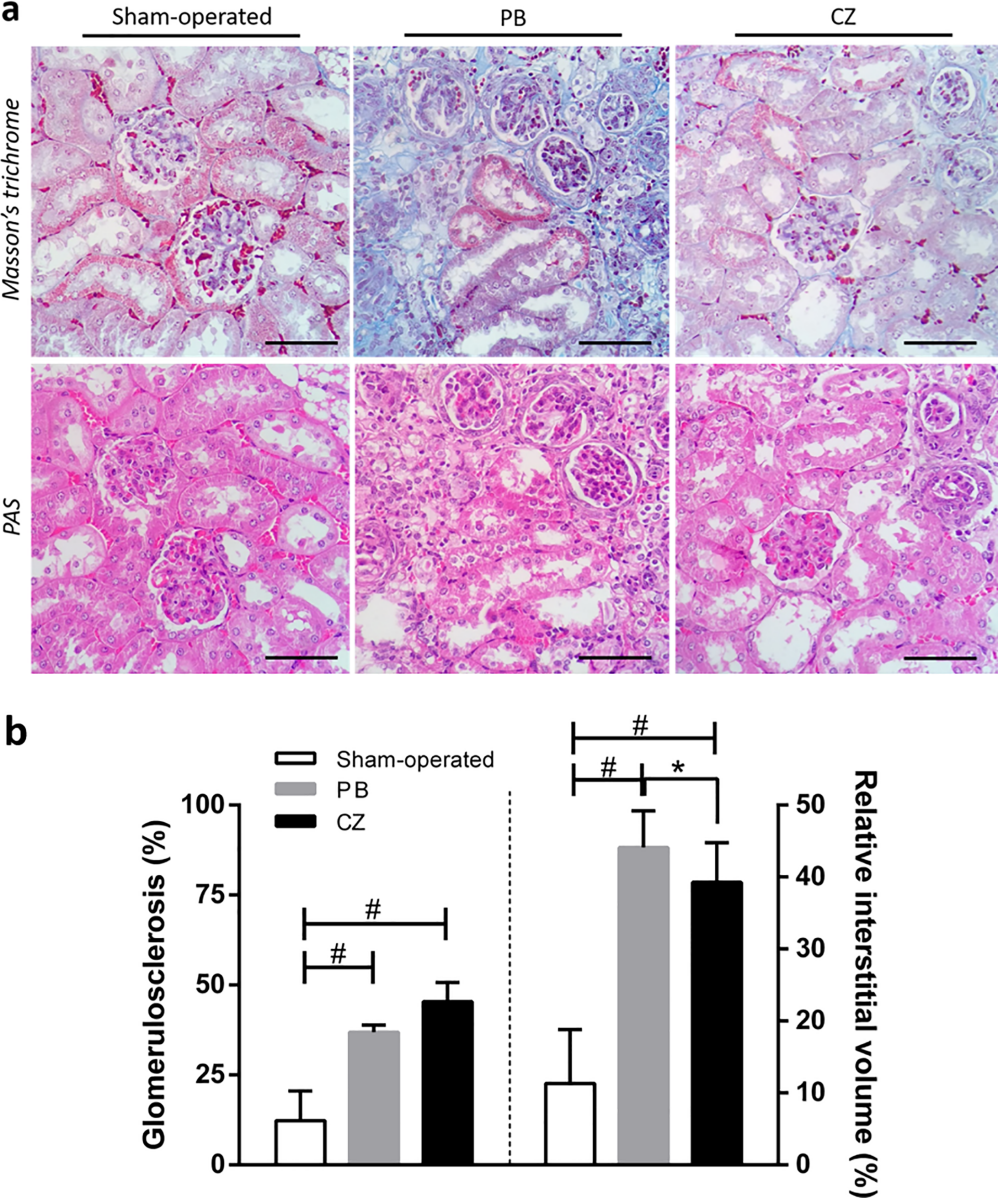
Renal pathology scoring at 20 weeks post-nephrectomy of the ischemic-reperfusion injury with unilateral nephrectomy (Chr I/R) mouse model. **(a)** The representative figures of renal cortical staining by Periodic Acid-Schiff (PAS), and Masson’s trichrome among sham-operated, placebo (PB), and cilostazol-treated (CZ) mice were demonstrated. **(b)** Glomerulosclerosis, the percentage of the glomerular area that was sclerotic determined from PAS-stained sections, (left-side panel) and relative interstitial volume, the percentage of total surface area of the sampled cortical area in Masson’s trichrome stained sections that is occupied by interstitial space (see method), (right-side panel) from Chr I/R in sham-operated (n = 6), placebo (PB) (n = 10), and cilostazol-treated mice (CZ) (n = 10) were demonstrated. *p< 0.05, #*p*< 0.0001. Scale bar = 200 μm.

Regarding kidney function, BUN and SCr increased as early as 2 weeks post-nephrectomy in both cilostazol-treated and placebo-treated mice. At baseline, 1 week before performing I/R injury in left kidney (wk-1), BUN and SCr were 14±2 and 0.3±0.1 mg/dL in cilostazol-treated mice, and 13±6 and 0.2±0.1 mg/dL in placebo-treated mice, respectively. At 20 weeks, post-nephrectomy, BUN and SCr progressed to 83±5 and 1.2±0.5 mg/dL in cilostazol-treated mice and 88±6 and 1.2±0.3 mg/dL in placebo-treated mice, respectively (Fig 2C). This finding conforms to the idea that the Chr I/R mice had gradual anemia as determined by the Hct reduction from the baseline (from 50±7% to 45±6%) 12 weeks post-nephrectomy. Ultimately, Hct at 20 weeks post-nephrectomy was 37±2% (Fig 2D). Despite progressive deterioration of kidney function, proteinuria did not differ between the sham-operated, placebo-treated and cilostazol-treated mice at 20 weeks (Fig 2E), suggesting the CKD model with predominant tubulointerstitial injury was proven by kidney pathology (Fig 3A). At 20 weeks, however, cilostazol-treated mice demonstrated lower relative interstitial volume than placebo-treated mice (*p*< 0.05) as determined by histopathology analysis (Fig 3B).

In addition, diuresis was showed in Chr I/R mice. Urine volume was 9±3 mL/day at 20 weeks after nephrectomy. Despite the diuresis in Chr I/R mice, 35% (7/20) of these mice showed frank pulmonary edema by gross examination. The mean of lung wet weight/body weight (LW/BW) ratio in Chr I/R mice was 10.4±3.9 mg/g compared to 3.3±0.1 mg/g in sham mice (*p*< 0.01).

### Cilostazol ameliorates vascular intimal hyperplasia at 20 weeks of ischemic-reperfusion injury with unilateral nephrectomy-induced chronic kidney disease (Chr I/R) mice

**Fig 4:**
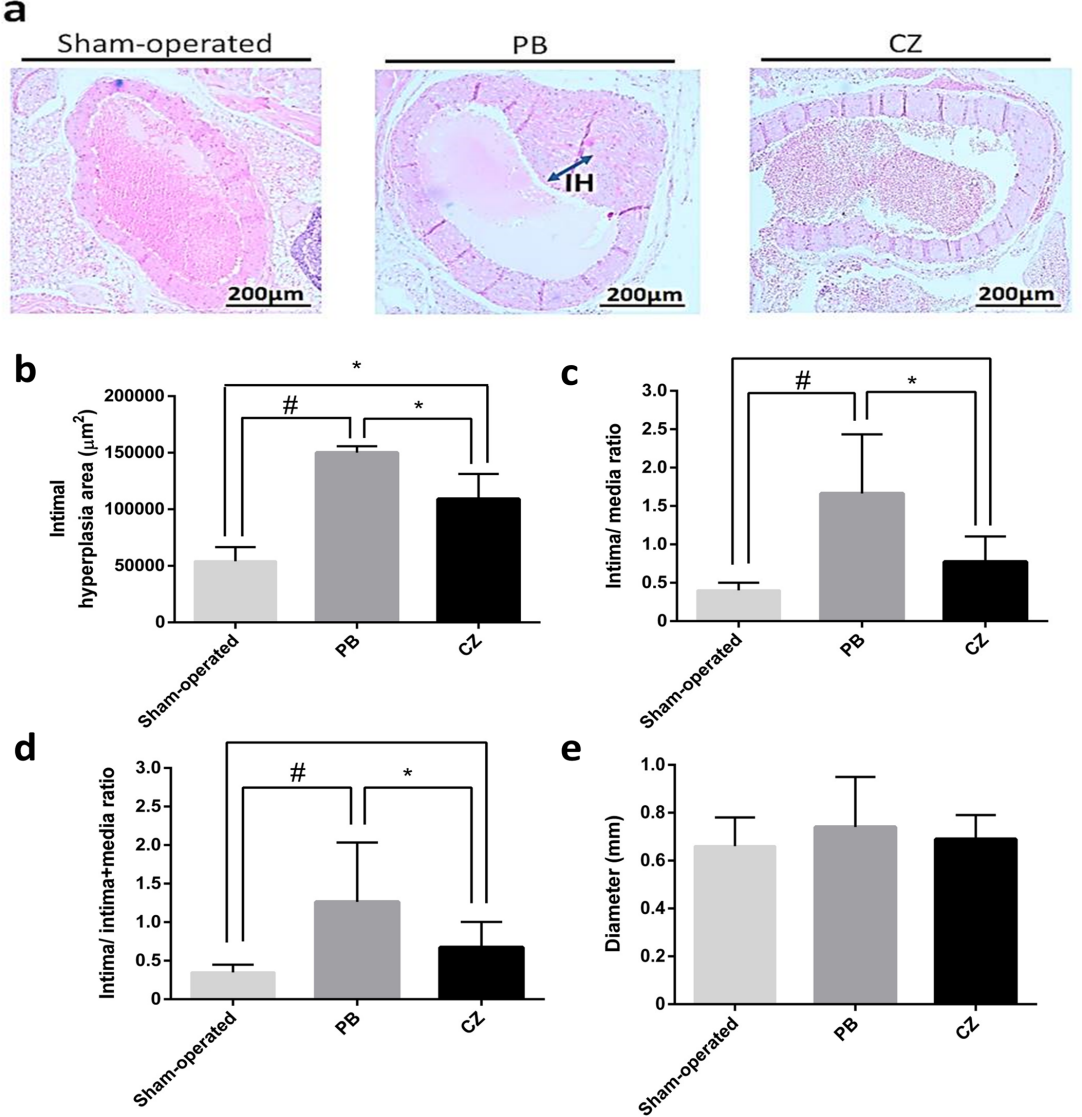
Aortic pathology at 20 weeks post-nephrectomy of the ischemic-reperfusion injury with unilateral nephrectomy (Chr I/R) mouse model. **(a)** The representative figures of aorta by H&E staining of sham-operated, placebo (PB) and cilostazol-treated mice (CZ) at 20 weeks post-nephrectomy were demonstrated. The semi-quantitative analysis regarding: **(b)** the average area of the IH area, **(c)** the ratio of tunica intima area/ tunica media area, **(d)** the ratio of tunica intima area/ (tunica intima area + tunica media area) and **(e)** aortic diameter of sham-operated (n = 6), placebo (PB) (n = 10), and cilostazol-treated mice (CZ) (n = 10) were demonstrated. *p< 0.01, #*p*< 0.0001.

Chr I/R mice demonstrated a positive correlation between aortic IH severity and relative interstitial volume (r^2^ = 0.74, *p* = 0.01) as well as BUN (r^2^ = 0.67, *p* = 0.004), but a weak correlation between IH severity and SCr (r^2^ = 0.12, *p*< 0.001) as shown in S1 Fig. Interestingly, cilostazol-treated mice showed a lower severity of IH (Fig 4A). At 20 weeks post-nephrectomy, IH in cilostazol-treated and placebo-treated mice was 99,704±22,310 and 150,556±5,669 μm^2^ (*p*< 0.01), respectively (S1 Table). These data are consistent with a 50% lower I:M ratio and a 46% lower I:I+M ratio. In contrast, the diameters of aorta of the cilostazol-treated group were not different from the placebo-treated group (Fig 4B–4E).

### Cilostazol effects on aortic tissue miRs, serum vascular smooth muscle cells (VSMCs)-related cytokines and inflammatory cytokines at 20 weeks of ischemic-reperfusion injury with unilateral nephrectomy-induced chronic kidney disease (Chr I/R) mice

**Fig 5:**
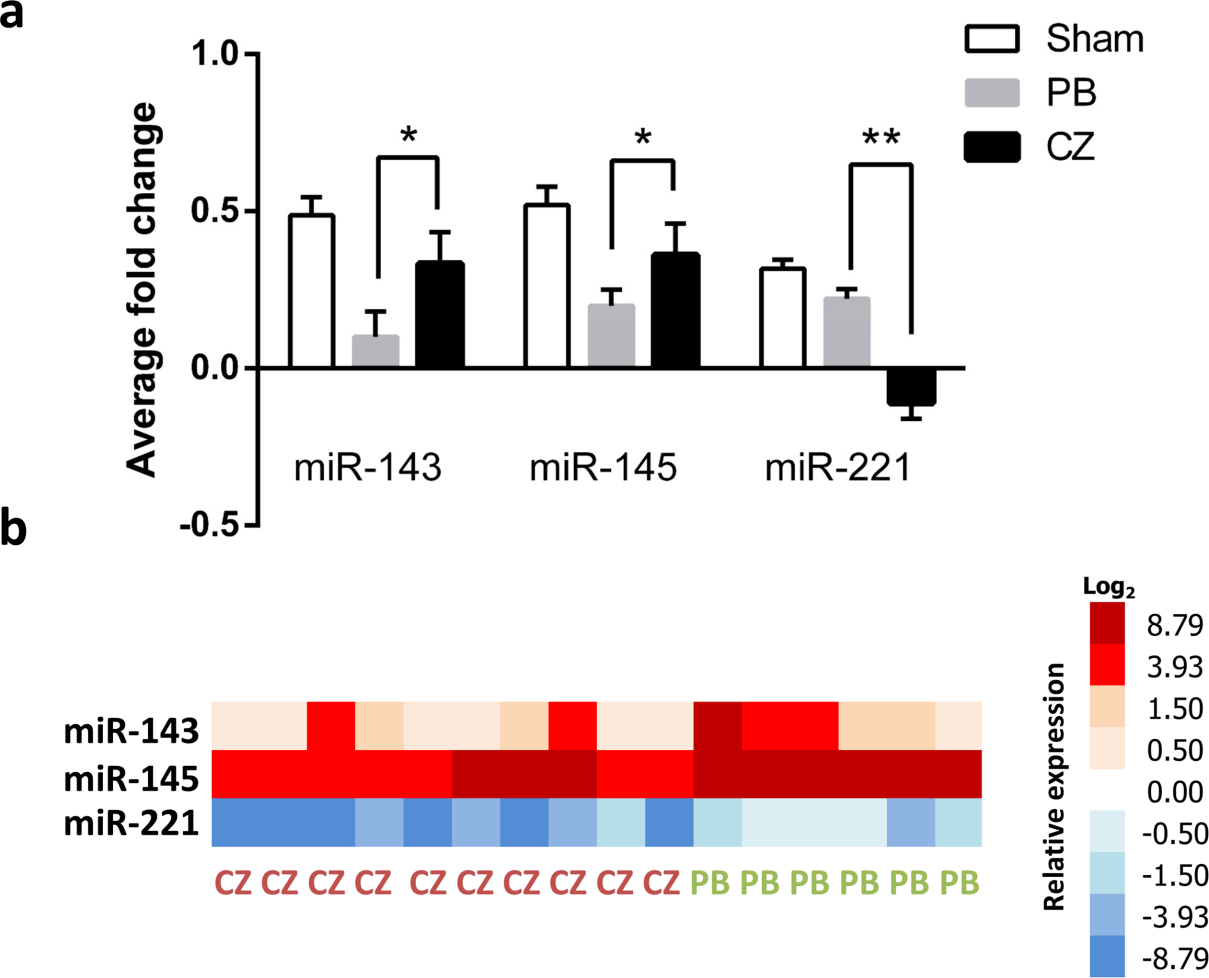
An analysis of microRNAs in aortic tissue at 20 weeks post-nephrectomy of the ischemic-reperfusion injury with unilateral nephrectomy (Chr I/R) mouse model. **(a)** MicroRNA in aortic tissue from sham-operated (n = 6), placebo (PB) (n = 10), and cilostazol-treated mice (CZ) (n = 10) at 20 weeks post-nephrectomy were showed. RNU48 was used for the normalization (see method). **(b)** A graphical heat-map presentation was demonstrated. Higher and lower ΔCt values were colored in red and blue, respectively; *p< 0.05, **p< 0.01.

**Fig 6:**
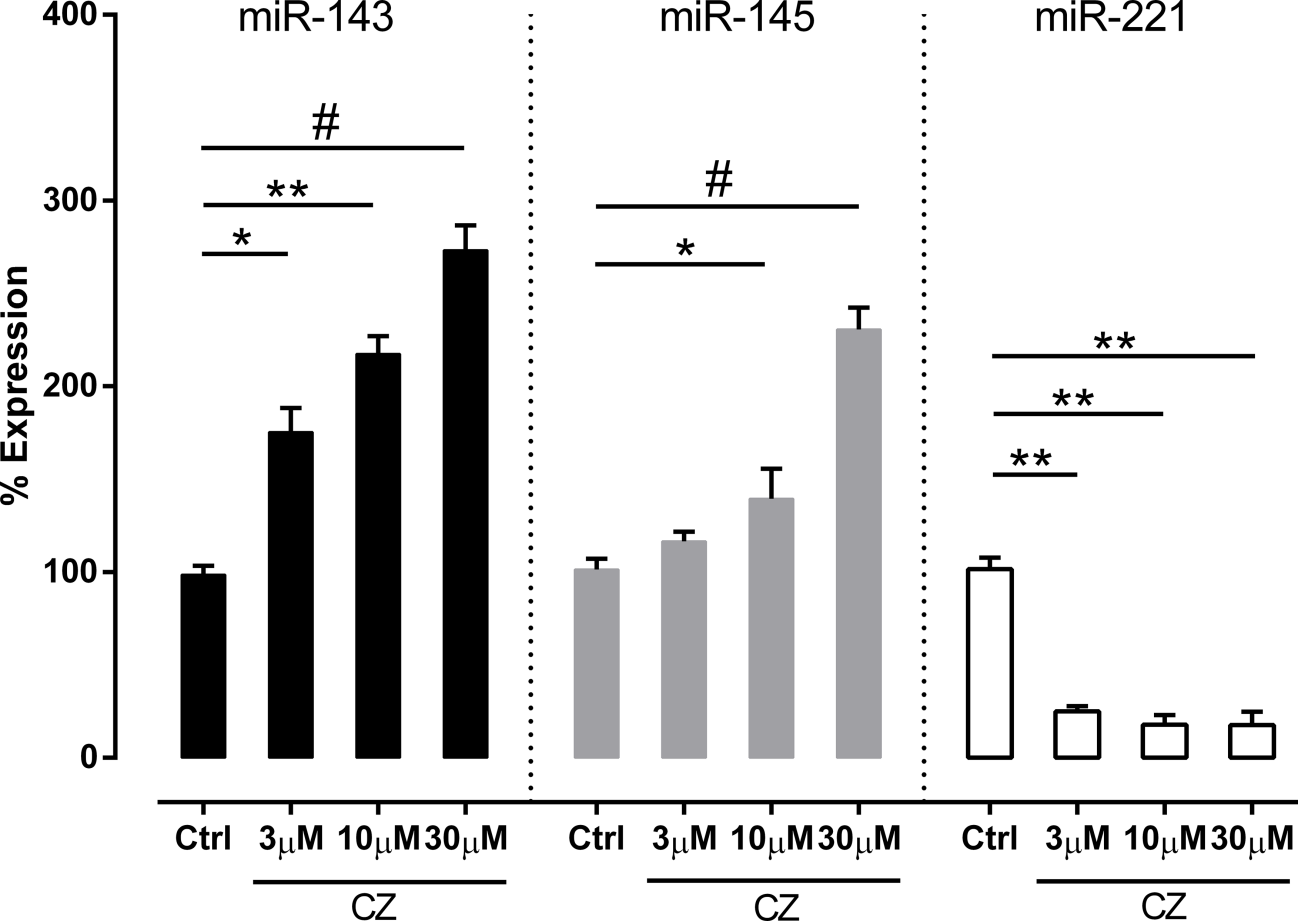
Effect of various concentrations of cilostazol on microRNAs (miRs) expression of human umbilical vein endothelial cells (HUVECs). HUVECs were incubated with 3, 10, 30 uM of cilostazol for 24 hours. The number of expression is presented as the percentage of expression determined by ΔΔCT and expression fold change relative to control. Data are represented as mean±standard error. *p< 0.05, **p< 0.01, and #*p*< 0.001 indicate significance relative to control. Sample size (n) = 3 for each group from 3 independent replicates. Ctrl, control (DMSO in EBM-2-treated) group; CZ, cilostazol-treated group.

Increased expression of miR-143 and miR-145 in aorta tissue was demonstrated in cilostazol-treated mice compared with the placebo control. On the contrary, miR-221 showed a significantly reduced expression in the cilostazol-treated group (Fig 5). To support the association between cilostazol and miR expression, an *in vitro* test on HUVECs was performed. As demonstrated in Fig 6, cilostazol significantly enhanced both miR-143 and miR-145 expression but reduced miR-221 expression in comparison with the control group.

**Fig 7:**
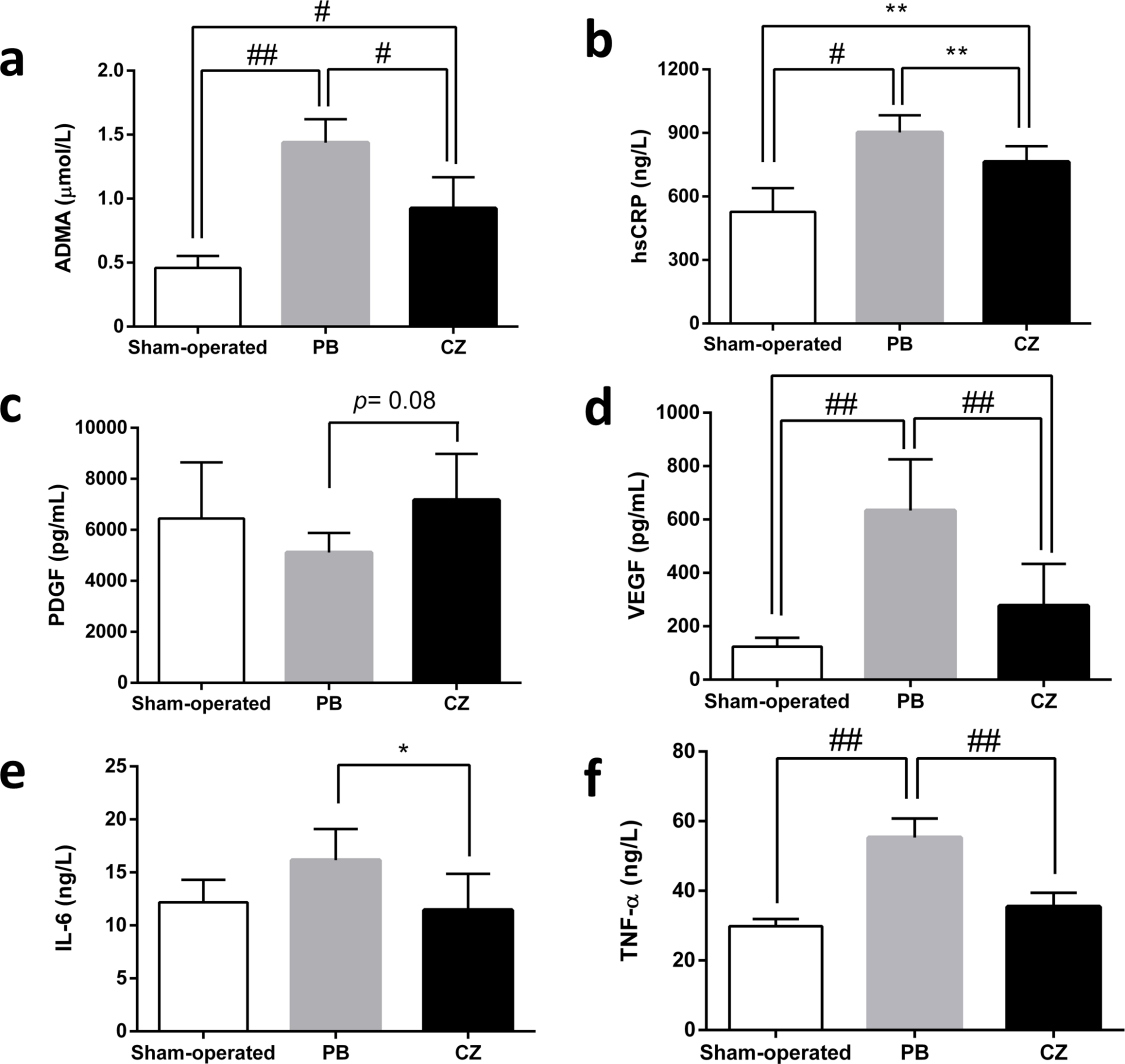
Serum concentration of several mediators at 20 weeks post-nephrectomy of the ischemic-reperfusion injury with unilateral nephrectomy (Chr I/R) mouse model. **(a)** asymmetrical dimethylarginine (ADMA), **(b)** high sensitivity C-reactive protein (hs-CRP), **(c)** platelet-derived growth factor (PDGF), **(d)** vascular endothelium growth factor (VEGF), **(e)** interleukin (IL)-6 and **(f)** tumor necrosis factor alpha (TNF-α) in sham-operated (n = 6), placebo (PB) (n = 10), and cilostazol-treated mice (CZ) (n = 10) at 20 weeks post-nephrectomy were demonstrated. *p< 0.05, **p< 0.01, #*p*< 0.001, ##*p*<0.0001.

Due to the influence of VSMC-related cytokines and pro-inflammatory processes in IH pathogenesis, we measured VEGF, PDGF, hs-CRP, IL-6, and TNF-α. In comparison with placebo-treated mice, there were significantly lower levels of serum VEGF, hs-CRP, IL-6, and TNF-α, but not serum PDGF, in cilostazol-treated mice. In addition, serum ADMA, a surrogate marker for vascular vasodilatory effect, was significantly lower in cilostazol-treated mice compared with the placebo group (Fig 7). Despite the effectiveness of cilostazol at 20 weeks on the attenuation of IH in Chr I/R mice, renal function as determined by BUN and SCr was not different between the cilostazol and placebo groups (data not shown).

**Fig 8:**
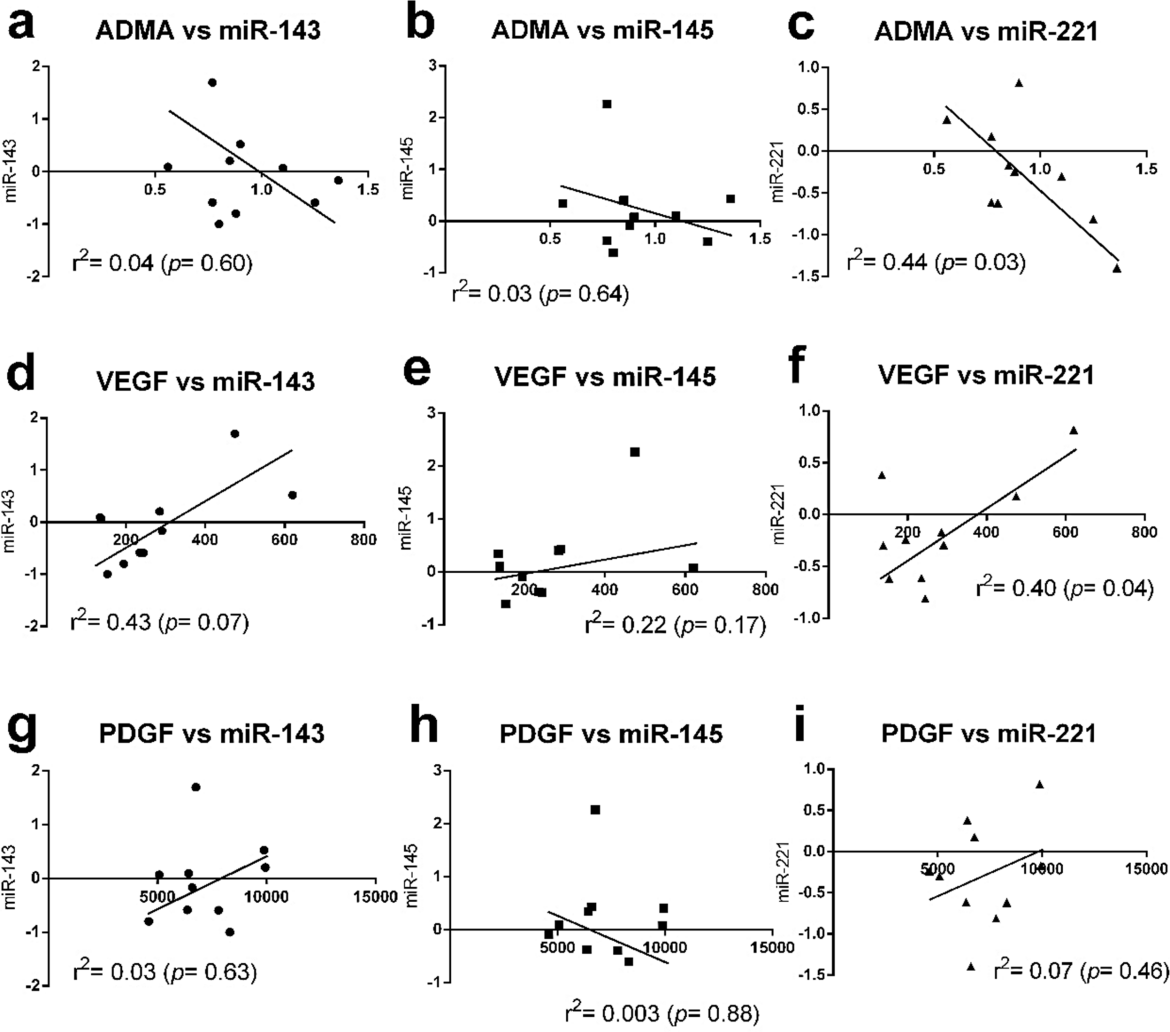
The correlation analysis between microRNAs (miRs) in aortic tissue and other serum parameters. The correlation between asymmetrical dimethylarginine **(**ADMA) and miR-143 **(a)**, miR-145, **(b)** miR-221 **(c)**; between vascular endothelium growth factor (VEGF) and these miRs **(d-f)** and between platelet-derived growth factor (PDGF) and these miRs **(g-i)** were demonstrated.

Because CKD increases pro-inflammatory mediators [29, 30] and the association between PDGF and VEGF with miRs in IH pathogenesis has been reported [12, 31, 32], we evaluated the concentration in sera over time to determine the cascade of these mediators in Chr I/R mice. We found that serum miR-221 was the earliest detectable biomarker among these molecules (detectable in 5 mice (50%) at 12 weeks after right nephrectomy). Subsequently, other molecules, including VEGF, miR-143, and miR-145, were detectable at 16 weeks (S2 Fig). Then, other serum biomarkers, including ADMA, PDGF, and inflammatory cytokines (IL-6, TNF-and hs-CRP), were demonstrated at 20 weeks after right nephrectomy (data not shown). Furthermore, the increase in expression of VEGF, hs-CRP, IL-6, and TNF-α in the Chr I/R mice was reversed in animals treated with cilostazol. Interestingly, a positive correlation was demonstrated between VEGF and miR-221, whereas a negative correlation was noted between ADMA and miR-221. There was no correlation observed between other parameters (Fig 8).

## Discussion

We demonstrated for the first time that cilostazol attenuates CKD-induced aortic IH in CKD with a Chr I/R mouse model. Cilostazol modulated several miRs responses, including reducing VEGF and ADMA. In addition, cilostazol also induced anti-inflammatory effects as demonstrated by the attenuation of several pro-inflammatory mediators (IL-6, TNF-α, and hs-CRP). These contributions support the usefulness of cilostazol as an adjunctive strategy for the prevention of CKD-induced IH.

### Arterial intimal hyperplasia and characteristics of Chr I/R model

Despite the diversity of IH from different diseases, the pathogenesis of IH can be explained through the direct and indirect endothelial injury processes [33, 34], which, for CKD, are hypertension and the metabolic consequences, respectively. Because direct-vascular-injury-induced IH is well-known, we aimed to investigate the predominantly indirect-vascular-injury effects of CKD on IH progression. Predominant tubulointerstitial damage with other CKD-related consequences, but with mild proteinuria, was demonstrated in Chr I/R in our study, supporting previous research [5]. Interestingly, although the protective effects of isoflurane on I/R injury has been demonstrated [24], both placebo- and cilostazol-treated mice developed CKD characteristics under its influence. Due to the minimized high blood pressure in this model, IH was possibly mainly due to indirect endothelial injury. Indeed, aortic IH of Chr I/R was demonstrated with a 30% increase in tunica intima thickness of all mice at 20 weeks post-nephrectomy. As such, a Chr I/R mouse model might be one of the most suitable CKD models for exploring indirect-endothelial-injury-induced IH. Interestingly, medial calcification in this CKD mice model was not prominent. We hypothesize that the calciphylaxis in this model is not as severe as in patients with CKD. This implies that the influence on calciphylaxis of calcium, vitamin D and/or diets in patients with CKD and in other *in vivo* models [35].

### Cilostazol attenuated chronic kidney disease-related IH, at least in part, through anti-inflammation and miRs modulation

The protective effects of cilostazol on CKD-related IH were demonstrated by the reduction of total tunica intima area, seen in the lower I:M ratio and I:I+M ratio in the cilostazol-treated mice compared with the placebo group. The lower I:M ratio implies that cilostazol was targeted toward the intima, while the lower I:I+M ratio supported the effectiveness of cilostazol on the intima layer. Although the attenuation of direct-endothelial-injury-induced IH by cilostazol has been previously demonstrated [36–38], this study shows for the first time the benefits on a CKD model with predominantly indirect-vascular-injury-induced IH. Our findings suggest that cilostazol might promote vascular protection predominantly through anti-inflammatory effects (the attenuation of hs-CRP, IL-6, and TNF-α) (Fig 7B, 7E and 7F). Perhaps the anti-inflammatory effects were important for the amelioration of the indirect-vascular-injury-induced IH, while the vasodilatory effects were important for the attenuation of the direct-vascular-injury-induced IH [39–41].

Although cilostazol theoretically induces VEGF, there was lower VEGF in cilostazol-treated Chr I/R [7, 42]. Indeed, VEGF has a paradoxical effect on endothelium. VEGF was induced by pro-inflammatory responses but important for the endothelial healing process [43]. As such, anti-VEGF is beneficial as an anti-inflammatory drug in pro-inflammatory states but disrupts the wound healing processes in a chronic injury model [44, 45]. The VEGF-induced endothelial proliferation might worsen IH. The lower VEGF in cilostazol-treated mice demonstrates the more prominent anti-inflammatory effects over the induction of endothelial proliferation, which is beneficial for IH attenuation.

On the other hand, circulating ADMA, a uremic toxin with endogenous NOS inhibitory effects and that induces pro-inflammatory responses [46], was also significantly decreased with cilostazol treatment. As observed in the rabbit model, ADMA inhibited NO, resulting in relative vasoconstriction, increased leucocyte and platelet adhesion, and enhanced IH [47]. As such, ADMA reduction after cilostazol treatment could also be beneficial. However, cilostazol did not attenuate PDGF, an important IH activator though VSMCs activation [48] in the Chr I/R model.

Furthermore, the association between miRs, VSMC regulation, and IH formation was recently demonstrated [10, 12]. Mice with the gene-deficiency of miR-143 and/or miR-145 showed prominent IH [49]; miR-145-blocking remarkably inhibited IH [12]; and a knockdown of miR-221 also inhibited IH [11]. We hypothesized that cilostazol might also mediate IH through miRs, and explored the expression of these miRs in aorta of Chr I/R mice. Indeed, we found that cilostazol inhibited tissue miR-221 but promoted tissue miR-143 and miR-145 in aorta and the role of miRs in IH pathogenesis [50]. In addition, the increased expression of miR-143 and miR-145 along with reduced miR-221 expression after cilostazol treatment were also demonstrated in HUVEC experiments.

Our data support previous studies on the importance of miR-221 [11], but demonstrate an opposite direction of miR-143 and miR-145 in association with IH severity [10, 12, 49]. These data imply diversity in IH pathogenesis in different models. Despite these data, the earliest time point of miR activation by cilostazol in relation to IH suppression was not defined in our study. Thus, although miR activation appeared to be a downstream event related to IH inhibition [10], the link between these 3 miR targets in CKD-related IH after Chr I/R requires further study.

Nevertheless, the correlation analysis demonstrated the association trends of miR-221 to VEGF and ADMA in a positive and a negative direction, respectively. Cilostazol decreases miR-221 together with VEGF. Because miR-221 induces IH through increased VEGF by Vegfc/Vegfr-3 signaling activation [51], it is possible that cilostazol may attenuate VEGF through decreased miR-221. We found that miR-221 had an early onset that decreased in concentration compared with VEGF, and miR-221 also had a higher percentage reduction from the baseline than VEGF at 16 weeks post-nephrectomy (93.5% and 71.6%, respectively, *p*< 0.05). On the other hand, cilostazol attenuated both miR-221 and ADMA, but there was an inverse trend between these parameters. This implies that both miR-221 and ADMA may be implicated in VSMC regulation; however, miR-221 may not be a differentially expressed miR for ADMA. Although an increasing body of evidence reveals the effects of miR221 and ADMA in early endothelial progenitor cells response [52, 53], their role in VSMCs is not well-known and is a fertile topic for future studies.

In conclusion, we demonstrated a proof of the effectiveness of cilostazol in attenuating IH in a Chr I/R mouse model, a CKD model with predominantly indirect-vascular-injury-induced IH. These considerations warrant further investigation to develop a new primary prevention strategy for CKD-related IH. The identification of tissue miR-221, ADMA, and inflammatory cytokines could also be interesting surrogate biomarkers to demonstrate the transition of biochemical changes into significant pathological defects. These data can open new avenues to manipulate vascular damage from IH.

## Supporting information

S1 Fig The correlation between aortic intimal hyperplasia severity and Chr I/R surrogate markers. **(a)** blood urea nitrogen (BUN), **(b)** creatinine (Cr) and **(c)** relative interstitial volume.
(TIF)

S2 Fig Percentage of mice with positive serum miRs, growth factors, and inflammatory cytokines after the ischemic-reperfusion injury with unilateral nephrectomy (Chr I/R) mouse model (n = 10). Serum miR-221 was demonstrated in 5 mice (50%) at 12 weeks after right nephrectomy. Subsequently, VEGF, miR-143 and miR-145 were detectable at 16 weeks of the model in 9 (90%), 7 (70%) and 7 (70%) mice, respectively. miR, miRNA; VEGF, vascular endothelial growth factor.
(TIF)

S1 Table Comparison of intimal hyperplasia area between cilostazol-treated and placebo-treated mice. (DOCX)
