# Supplementary material for: Cilostazol attenuates intimal hyperplasia in a mouse model of chronic kidney disease
Source: PLoS One. 2017 Dec 5;12(12):e0187872. doi: 10.1371/journal.pone.0187872 (PMC5716535; doi:10.1371/journal.pone.0187872)
Supplement: S1 Table — (DOCX) [file pone.0187872.s003.docx]

**S1 Table.** Comparison of intimal hyperplasia area between cilostazol-treated and placebo-treated mice.

| **Animal No.** | **Intimal hyperplasia area (μm2)** | |
| --- | --- | --- |
|  | **Cilostazol-treated mice** | **Placebo-treated mice** |
| 1 | 124,423 | 150,915 |
| 2 | 99,905 | 155,470 |
| 3 | 75,984 | 154,103 |
| 4 | 70,921 | 142,536 |
| 5 | 98,019 | 157,222 |
| 6 | 133,250 | 153,620 |
| 7 | 82,127 | 150,319 |
| 8 | 129,365 | 147,963 |
| 9 | 87,520 | 139,725 |
| 10 | 95,529 | 153,695 |
